# A Novel Ferroptosis-Related Gene Model for Overall Survival Predictions of Bladder Urothelial Carcinoma Patients

**DOI:** 10.3389/fonc.2021.698856

**Published:** 2021-07-27

**Authors:** Min Zhang, Xin Zhang, Minghang Yu, Wei Zhang, Di Zhang, Song Zeng, Xi Wang, Xiaopeng Hu

**Affiliations:** ^1^Department of Urology, Beijing Chao-Yang Hospital, Capital Medical University, Beijing, China; ^2^Institute of Urology, Capital Medical University, Beijing, China; ^3^Department of Immunology, School of Basic Medical Sciences, Advanced Innovation Center for Human Brain Protection, Beijing Key Laboratory for Cancer Invasion and Metastasis, Department of Oncology, Capital Medical University, Beijing, China; ^4^Department of Urology, First Hospital of Shanxi Medical University, Taiyuan, China

**Keywords:** ferroptosis, bladder neoplasms, prognostic model, nomogram, TCGA

## Abstract

**Introduction:**

Bladder cancer is the most common urinary tract malignancy, and 90% of bladder tumors are urothelial cell carcinomas. Ferroptosis is a new form of cell death discovered in recent years, which is an iron-dependent form of cell death characterized by the lethal intracellular accumulation of lipid-based reactive oxygen species. Ferroptosis is considered to be a double-edged sword for cancer and cancer therapy.

**Materials and Methods:**

In the current study, expression profiles of bladder cancer (BLCA) specimens were obtained from The Cancer Genome Atlas (TCGA) RNA-Seq database. Ferroptosis-related genes were downloaded from the FerrDb website. The ferroptosis-related differentially expressed genes (DEGs) which were related to overall survival (OS) were first identified. The least absolute shrinkage and selection operator (LASSO) and multivariate Cox regression methods were utilized to develop a ferroptosis-related prognostic model (FRPM). In addition, a nomogram model based on FRPM and clinicopathological features was successfully constructed and validated. In addition, gene ontology (GO), Kyoto Encyclopedia of Genes and Genomes (KEGG), and single-sample gene set enrichment analysis (ssGSEA) methods were utilized in this study in order to compare the DEGs between the high-risk and low-risk groups. This study also adopted RT-qPCR, CCK-8 assay, and scratch assay methods to perform experimental verification processes.

**Results and Discussion:**

A 7-gene FRPM was constructed in this research investigation in order to stratify the patients into two groups according to their risk scores. The results of this study’s survival analysis and time-dependent receiver operating characteristic (ROC) analysis demonstrated that the model had achieved a stable performance level. This multivariate Cox regression results revealed that the FRPM was an independent prognostic predictor for the OS of BLCA patients and the results were displayed using a nomogram. In addition, the ROC analysis, concordance index (C-index), calibration plots, and decision curve analysis (DCA) curves further indicated that this study’s nomogram method enabled valuable prediction results. The functional enrichment analysis results suggested that the DEGs between the high- and low-risk groups played vital roles in the progression of the ferroptosis. Also, the ssGSEA indicated that the immune status was different between the two groups. This study found that the RT-qPCR results had confirmed the differential expressions of DEGs in the tissue samples, and the CCK-8 assay and scratch assay results confirmed the promoting effects of SCD on the proliferation and migration of tumor cells.

**Conclusions:**

This study defined a novel prognostic model of seven ferroptosis-related genes, which proved to be independently associated with the OS of BLCA. A nomogram method was developed for the purpose of providing further insight into the accurate predictions of BLCA prognoses.

## Introduction

Bladder cancer (BLCA) is the most common malignancy of the urinary tract originating from bladder mucosa. BLCA ranks as the ninth most frequently diagnosed cancer worldwide (1). It has been found that 90% of bladder tumors are urothelial cell carcinomas (2). Smoking, as well as exposure to occupational environments containing aromatic amines, PAHs, and other chemicals, are the most prominent risk factors for bladder cancer incidence (3, 4). The malignancy characteristics of bladder cancer, particularly the levels of invasiveness, are closely related to their recurrence rates and long-term survival rates. Numerous staging grading systems have been proposed in order to assess bladder tumor prognoses. Among those, the most widely used is the tumor node metastasis (TNM) classification system. However, there has been evidence that patients of the same pathological grade or clinical stage of bladder cancer may still have different prognoses and survival rates, which suggests that the presence of factors other than those listed in the existing TNM classification systems may influence patient outcomes (5). Moreover, in recent years, several studies have investigated bladder tumor prognosis-related genes and constructed survival prediction models for bladder tumors based on such database information as The Cancer Genome Atlas (TCGA) (5–7).

In recent years, a new form of cell death referred to as ferroptosis was discovered. Ferroptosis is an iron-dependent form of cell death characterized by lethal intracellular accumulations of lipid-based reactive oxygen species (8). It has been found to play an important role in several disease processes, including cancer, and has gradually become a research hotspot in the field of programmed cell death (9). Many previous studies have explored the associations between ferroptosis and cancer development and multiple antitumor treatment modalities (10). Generally speaking, ferroptosis is a double-edged sword for cancer and cancer therapy (11). On one hand, ferroptosis is involved in the process of radiotherapy, chemotherapy, targeted therapy, immunotherapy, and so on. The activation of ferroptosis progression in cancer cells can exert antitumor effects (12). On the other hand, cancer cells undergoing ferroptosis can release specific signaling molecules which suppress tumor immune cells or upregulate immune checkpoints to allow other tumor cells to escape immune surveillance and continue growing (13).

In terms of urinary neoplasms, some previous studies have reported associations between ferroptosis and specific types of urinary cancer, such as kidney cancer and prostate cancer (14–17). However, the impacting effects of ferroptosis on bladder cancer have not yet been addressed. This study defined a novel prognostic model of seven ferroptosis-related genes, which proved to be independently associated with the overall survival (OS) rates of BLCA.

## Materials and Methods

### Data Acquisition Process

In the present investigation, the level 3 RNA sequencing data and corresponding clinical information for 411 BLCA samples and 19 normal samples were retrieved from the TCGA database (https://portal.gdc.cancer.gov/) on March 1^st^ of 2021.

The ferroptosis-related genes (FRGs) were downloaded from the FerrDb website (http://www.zhounan.org/ferrdb/), which is a database containing ferroptosis regulators and markers (**Supplementary Table S1**). Then, after the removal of duplicate genes, a total of 259 genes were successfully identified.

### Construction and Validation of a Prognostic Ferroptosis-Related Gene Model

**Figure 1:**
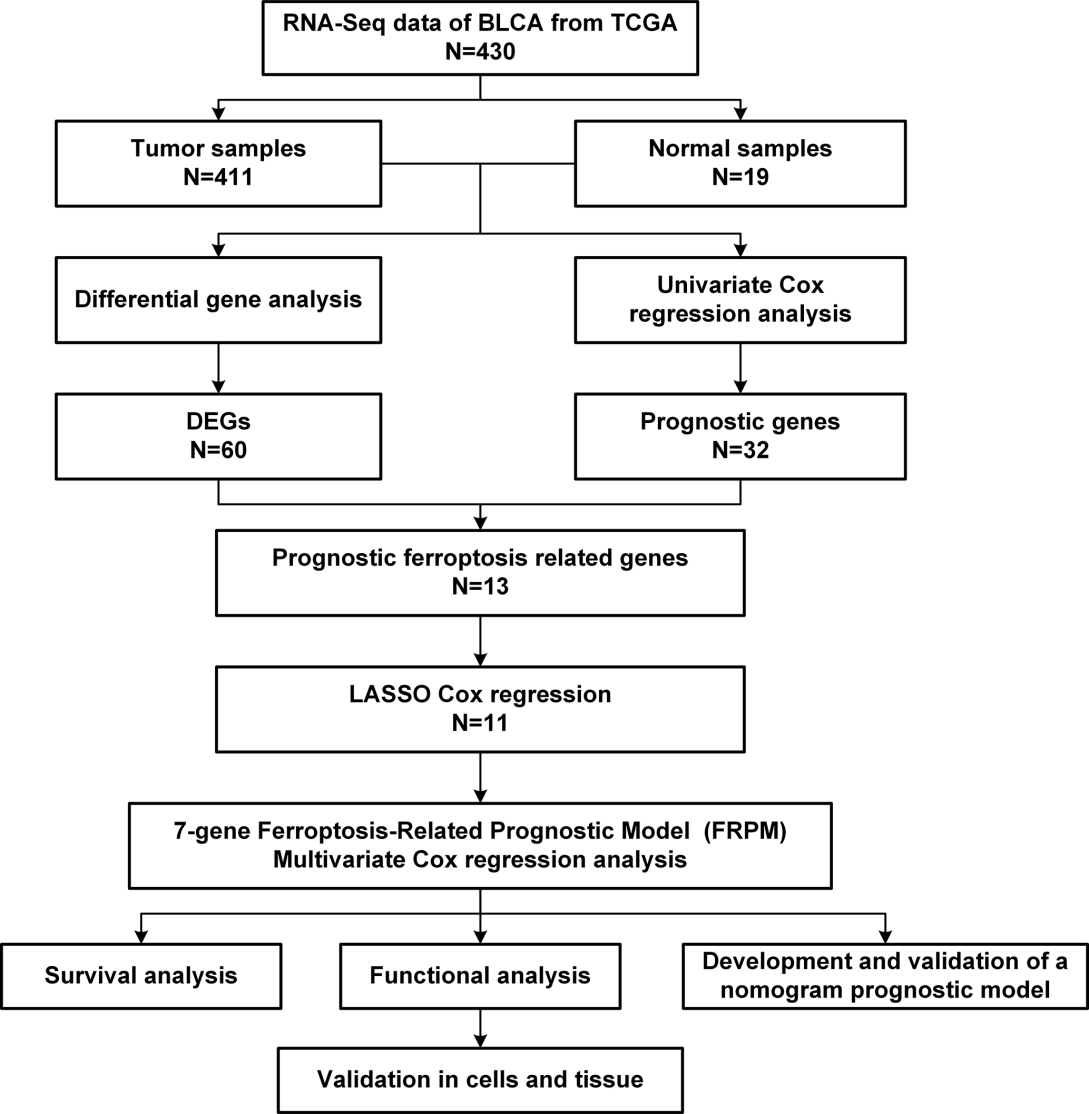
Flow chart of the data collection and analysis processes.

This study’s workflow is illustrated in **Figure 1**. First of all, a “limma” R package was used to identify the ferroptosis-related differentially expressed genes (DEGs) among the BLCA samples and adjacent normal tissues with a threshold set as |log2 (Fold Change [FC])| > 0.5 and a false discovery rate (FDR) < 0.05. Then, after removing samples without complete survival information and samples with follow-up times less than 30 days, this study evaluated the associations between the ferroptosis-related DEGs and patients’ survival rates using a univariate Cox proportional hazards regression model (18).

At that point, ferroptosis-related DEGs with P < 0.05 were selected into the least absolute shrinkage and selection operator (LASSO) penalized Cox regression analysis using a “glmnet” R package in order to minimize the risks of overfitting (19, 20). The optimal value of the penalty parameter (lambda) was considered to be the minimum, as selected by a tenfold cross-validation process. Subsequently, a risk score model was constructed using the regression coefficients in the multivariate Cox regression analysis results. Then, a formula was established as follows:

$$\text{Risk Score}=\Sigma_{i\;=\;1}^{n}(\text{Coef}\times \text{exp}),$$

where N, Coef, and exp represented the number of genes and regression coefficients obtained in the previous step and expression level of the related gene, respectively.

The examined patient profiles were classified into high- and low-risk groups using a median risk score splitting process. Subsequently, Kaplan-Meier survival analysis was completed and the time‐dependent receiver operating characteristic (ROC) curves were determined, and a “survminer” R package and a “survival ROC” R package, respectively, were used to assess the accuracy and validity of the model. In addition, principal component analysis (PCA) and t-distributed stochastic neighbor embedding (t-SNE) analysis were used to perform dimension reductions using a “stats” R package and a “Rtsne” R package, respectively.

### Relationships Between the FRPM and the Traditional Clinical Characteristics

Any samples without complete clinical information were first removed (age, gender, grade, stage, and so on). Then, the correlations between the risk scores and the clinicopathological characteristics were analyzed using the Fisher’s exact probability test. The results were displayed as a heatmap. Subsequently, univariate and multivariate Cox regression analyses were performed for the purpose of assessing the survival predictive performances of those clinical features and risk scores. In the present investigation, SPSS23.0 statistical software (IBM Corporation, Armonk, NY) was used to complete the statistical analysis and forest plots were plotted using GraphPad Prism 8.0.1 software (GraphPad Software, San Diego, CA).

### Development and Validation of the Nomogram

A series of clinical indicators were determined, including age, sex, and stage, based on the results of the multivariate Cox analysis and FRPM in order to construct a novel nomogram for the prediction of patient survival rates in a clinical setting. The discriminant ability of the nomogram was assessed by the concordance index (C-index), which was corrected by 1,000 resamples using a bootstrap method. The time-dependent ROC curves were plotted for the purpose of evaluating the model’s predictive performance. In addition, the calibration curves were used to assess the agreement between the model-predicted risks and the actual risks. In addition, a decision curve analysis (DCA) method was employed in order to evaluate the application prospects and clinical utility potential of the nomogram.

### Functional Enrichment Analysis

A “limma” R package was used to identify the DEGs between the high- and low-risk groups, with the threshold set as |log2 (FC)| > 1 and FDR < 0.05. Gene ontology (GO) and the Kyoto Encyclopedia of Genes and Genomes (KEGG) were explored using a “cluster profiler” R package based on the DEGs. Then, a “ggplot2” R package was implemented in order to complete the visualization of the results. Finally, single-sample gene set enrichment analysis (ssGSEA) was performed in order to assess the infiltrating scores of the different immune cell subsets and immune-related functions among the high- and low-risk groups (21). The annotated gene set file was provided in this study’s **Supplementary Table S2** (22).

### Clinical Specimens

Bladder cancer tissue samples and paired normal samples were selected using the following criteria:

1. The patients had been diagnosed with bladder cancer and undergone surgery in the Beijing Chao-Yang Hospital; 2. The final diagnoses of the samples were muscle-invasive urothelial carcinoma; 3. The patients had not received any preoperative treatments. 

### Cell Cultures and Reagents

The human bladder cancer cell line J82 cells were cultured in MEM Alpha Medium (Gibco, USA). The RT4 cells and T24 cells were maintained in McCoy’s 5A Medium (iCell, China), and the 5637 cells were cultured in RPMI1640 Medium (iCell, China). The UMUC3 cells and TCCSUP cells were cultured in MEM (iCell, China), and the SW780 cells were maintained in L15 Medium (iCell, China). The human urothelial cells line SV-HUC-1 cells were cultured in F12K medium (iCell, China). All of the mediums were supplemented with 10% fetal bovine serum (FBS, BI, ISR), 100 μg/mL of penicillin, and 100 U/mL of streptomycin (Invitrogen). All of the examined cells were grown at 37°C with 5% CO_2_.

The SCD inhibitor (A939572) utilized in this study was purchased from MedChem Express (HY-50709).

### RNA Extraction and RT-qPCR Methods

The total RNA was extracted using a FastPure Cell/Tissue Total RNA Isolation Kit (Vazyme, China) following the manufacturer’s instructions. Then, the reverse transcription reactions were performed using HiScript III RT SuperMix for qPCR (+gDNA wiper) (Vazyme, China) in accordance with the instructions provided. Next, qPCR was completed using an AceQ Universal SYBR qPCR Master Mix (Vazyme, China).

The relative expression levels of the genes were analyzed using the comparative *Ct* method:

[*delta*][*delta*]*Ct*  =  [*delta*]*Ct*, sample − [*delta*]*Ct*, GAPDH.

The specific primers for the target genes were listed in **Table 1**.

**Table 1:** Primer sequences of related genes for RT-qPCR.

Gene		Sequence (5’-3’)
TP63	F*	GTCATTTGATTCGAGTAGAGGGG
	R	CTGGGGTGGCTCATAAGGT
SLC2A12	F	CTTGCCTCACTCACCGGAG
	R	GCGTCCCACTATAAGAACCGTG
CAPG	F	GCAGCTCTGTATAAGGTCTCTGA
	R	TTTCGCCCCTTCCAGATATAGA
TXNIP	F	ATATGGGTGTGTAGACTACTGGG
	R	GACATCCACCAGATCCACTACT
MAFG	F	CCTCAGAGAACGCCAGCA
	R	CCACCTTGCCTGGGACGA
SCD	F	TTCCTACCTGCAAGTTCTACACC
	R	CCGAGCTTTGTAAGAGCGGT
ZNF419	F	GCCTCTCTGGGACTTGCATC
	R	CATGCCAACAACCTCTCGGTA
GAPDH	F	AAGAAGGTGGTGAAGCAGGC
	R	GAGTGGGTGTCGCTGTTGAA

*F, forward; R, reverse.

### Cell Viability Assay Method

The J82 and RT4 cells were seeded into 96-well plates at a density of 2,000 cells per well. After cell attachment, SCD inhibitor (A939572) was added at concentrations of 5 and 10 nM. The cell viability was measured at various time points (for example, 0, 24, 48, and 72 hours) using a Cell Counting Kit-8 (CCK-8) (Vazyme, China). Then, 100 μL of culture medium containing 10 μL of CCK-8 solution were incubated in each well at 37°C for two hours under dark conditions. The absorbance was measured under 450 nm using a microplate reader (BioTek, USA).

### Scratch Assay Method

In this study, J82 and RT4 cells in the logarithmic growth phase were seeded into 6-well plates at a density of 1×10^6^ cells per well and cultured for 12 to 14 hours. Wounds were made with a sterile plastic 200 µl pipette tip after the cells reached over 90% confluence. Then, the cells were washed 2 to 3 times with PBS in order to remove any cellular debris, and fresh serum-free medium containing different concentrations of A939572 (for example, 0, 5, and 10 nM) were applied. The cells were observed and photographed at 0, 6, and 24 hours after scratching. The experiments were performed in triplicate and the scratch areas were calculated using Image J software. The cell migration rates were calculated using the following formula:

Migration rate (%)=(original distance−measured distance)/original distance×100%.

## Results

### Identification of the Prognostic Ferroptosis-Related Differentially Expressed Genes

In the present study, based on the filtering criteria of the DEGs, 60 ferroptosis-related DEGs were obtained between 411 BLCA samples and 19 adjacent normal samples, including 36 upregulated genes and 24 downregulated genes.

**Figure 2:**
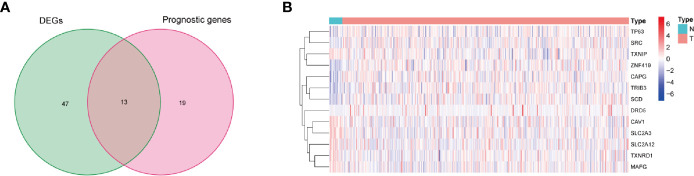
Identification of prognostic ferroptosis-related differentially expressed genes. **(A)** Venn diagram for identifying ferroptosis-related DEGs related to OS. **(B)** Expressions of thirteen overlapping genes, in which the upregulated and downregulated DEGs were indicated in red and blue, respectively, and N and T represented adjacent normal samples and tumor samples, respectively.

Then, in order to further explore the associations between the ferroptosis-related DEGs and the overall survival rates of the BLCA patients, this study obtained 13 prognostic ferroptosis-related DEGs (P < 0.05) using a univariate Cox regression analysis method, as detailed in **Figure 2A** and **Supplementary Table S3**. The results were visualized in the form of a heat map, as shown in **Figure 2B**.

### Construction of the FRPM

**Figure 3:**
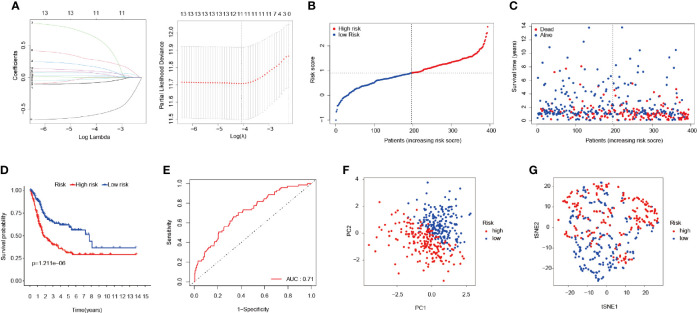
Construction of the FRPM. **(A)** LASSO Cox regression model applied to screen key genes, and partial likelihood deviance with 10-fold cross-validation was utilized to calculate the best lambda. **(B)** Distributions and median value of the risk scores in BLCA patients. **(C)** Distributions of the survival status values. **(D)** Kaplan-Meier curves for the seven genes relative to the overall survival outcomes. **(E)** AUC of the time-dependent ROC curve showing the predictive efficiency. **(F, G)** PCA plot and t-SNE analysis.

LASSO regression was applied for the purpose of screening the key genes among the ferroptosis-related DEGs, which were observed to be significant in the univariate Cox regression analysis results (**Figure 3A**). Then, based on the optimal value of λ, eleven genes were selected for the following multivariate Cox regression analysis process, and their respective relative coefficients were calculated. Finally, seven DEGs (TP63, SLC2A12, CAPG, TXNIP, MAFG, SCD, and ZNF419) were identified in order to establish the FRPM based on the risk scores. The risk score of each patient was calculated as follows: risk score = (-0.11379 × the expression level of TP63) + (0.23655 × the expression level of SLC2A12) + (0.13103 × the expression level of CAPG) + (-0.12909 × the expression level of TXNIP) + (0.38070 × the expression level of MAFG) + (0.14754 × the expression level of SCD) + (-0.68462 ×the expression level of ZNF419).

At that point, the patients were divided into a high-risk (n = 197) group and a low-risk group (n = 197) according to a median risk score, as detailed in **Figure 3B**. The patients in the high-risk group had a remarkably higher mortality rate than those in the low-risk group (**Figure 3C**). As can be seen in **Figure 3D**, the Kaplan-Meier survival curves illustrated that the high-risk patients had significantly lower survival rates when compared with the patients in the low-risk group (P < 0.001). Furthermore, a time-dependent ROC curve was applied in order to assess to prognostic prediction ability of the FRPM in BLCA patients. The area under the ROC curve (AUC) was 0.71 (**Figure 3E**), which demonstrated that the model had achieved a stable performance. In addition, the BLCA patients within the different risk groups were distributed in two directions using PCA and t-SNE analysis methods, as shown in **Figures 3F, G**.

### Independent Prognostic Values of the FRPM

**Figure 4:**
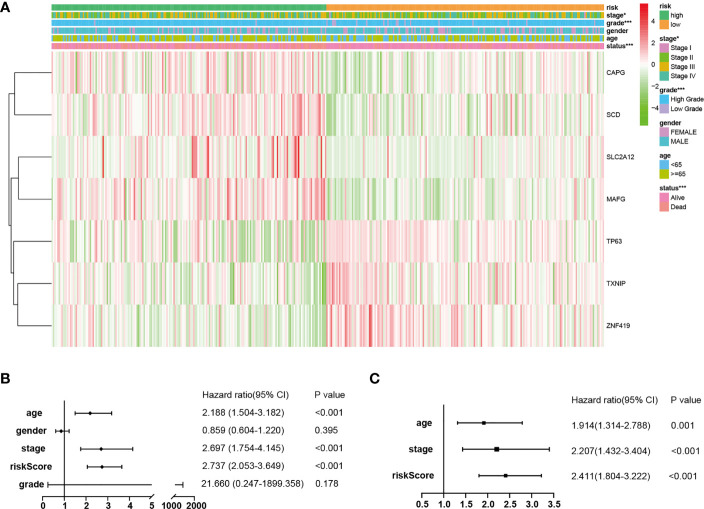
Independent prognostic values of the FRPM. **(A)** Correlations between the risk groups and the clinical traits. **(B, C)** Univariate and multivariate regression analysis results of the relationships between the FRPM and the clinical features. *P < 0.05, ***P < 0.001.

The results of the correlation analysis between the risk groups and the clinicopathological features were obtained using the Fisher’s exact probability test and visualized in the form of a heat map (**Figure 4A**). From the above-mentioned analysis results, it was determined that the risk groups had strong correlations with the tumor stages (P = 2.039E-02), tumor grades (P = 5.487E-04), and survival status (P = 8.907E-07). However, they were not observed to be related to age (P = 0.061) or gender (P = 0.301). In the high-risk group, the expression levels of CAPG, SCD, SLC2A12, and MAFG were found to be markedly upregulated, while expression levels of TP63, TXNIP, and ZNF419 were greatly downregulated (**Figure 4A**).

Therefore, in order to identify whether the FRPM was an independent prognostic predictor for OS, univariate and multivariate Cox regression analyses were performed for the purpose of examining the variables of the BLCA patients. The univariate Cox regression results revealed that the risk scores were strongly related to the OS [Hazard ratio (HR): 2.737, 95% CI (2.053–3.649), P < 0.001; **Figure 4B**]. It was observed that even after adjusting the other clinical features in the multivariate Cox regression, the risk scores had remained as prognostic values [Hazard ratio (HR): 2.411, 95% CI (1.804–3.222), P < 0.001; **Figure 4C**].

### Development and Validation of the Prognostic Nomogram Model

**Figure 5:**
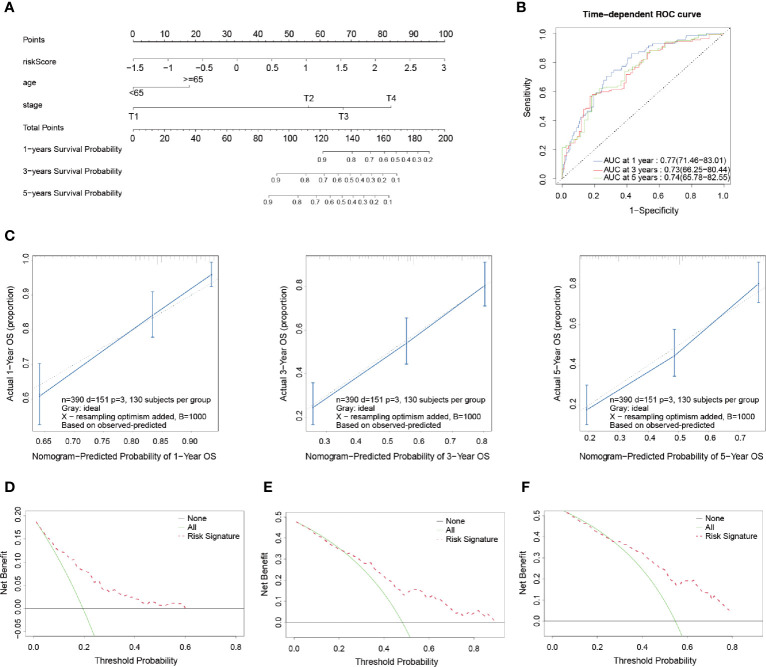
Development and validation of a prognostic nomogram model. **(A)** Nomogram for predicting the probability of 1-, 3-, and 5-year OS. **(B)** Time-dependent ROC curves of the nomogram. **(C)** Calibration curves of the nomogram for predicting the probability of OS in 1-, 3-, and 5-year timeframes. **(D–F)** Decision curve analysis for the OS in BLCA patients at 1, 3, and 5 years, respectively.

A nomogram is an intuitive, image-based, and practical tool which offers a convenient and straightforward way to improve clinical applications. Therefore, this study integrated independent clinical prognostic factors based on the results of the multivariate Cox regression analyses in order to develop a nomogram model for predicting 1-, 3- and 5-year survival probability rates (**Figure 5A**). By making a vertical line upward to the score scale line at the top, each variable could obtain its own corresponding score. Then the predicted probability rates were calculated by summing up all of the scores.

In order to evaluate the prognostic performance of the constructed survival nomogram, the time-dependent ROC curves were plotted for the purpose of predicting the survival rates at the 1-, 3- and 5-year timeframes (**Figure 5B**). The C-index was 0.73 (95% CI: 0.71–0.75), which also indicated the strong discrimination ability of the nomogram. In addition, the calibration plots revealed a good agreement between the actual probabilities and the estimated probabilities at 1, 3 and 5 years, as shown in **Figure 5C**. Moreover, DCA was performed, which showed that the predictions of survival probability when this study’s model was applied would be better than having all patients or none of the patients’ survival rates predicted by this model, with a range of the threshold probability between > 1% and < 60% at 1 year (**Figure 5D**); between >1% and < 85% at 3 years (**Figure 5E**); and between > 5% and < 80% at 5 years (**Figure 5F**), respectively.

### Functional Enrichment Analyses

**Figure 6:**
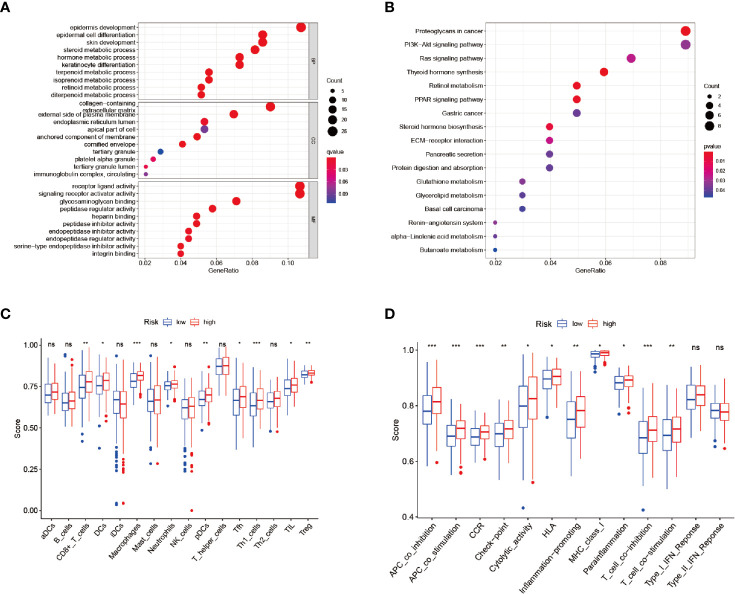
Enrichment analysis and ssGSEA scores. **(A, B)** GO and KEGG analyses. **(C, D)** Comparison of the ssGSEA scores between the different risk groups: Scores of the 16 immune cells and Scores of the 13 immune-related functions. *P < 0.05, **P < 0.01, ***P < 0.001, ns, no significance.

Functional enrichment analyses were conducted in order to further elucidate the underlying biological functions and pathways of the DEGs between the high-risk and low-risk groups, including GO and KEGG analyses. The GO analysis results (**Figure 6A**) revealed that the DEGs mainly focused on several metabolic processes, such as steroid metabolic processes, terpenoid metabolic processes, isoprenoid metabolic processes, diterpenoid metabolic processes, and so on. Moreover, the KEGG functional enrichment analysis results (**Figure 6B**) indicated several specific categories, such as steroid hormone biosynthesis, glutathione metabolism, glycerolipid metabolism, and so on, were significantly enriched in those DEGs. The results demonstrated that the DEGs between the high- and low-risk groups played vital roles in the progression of ferroptosis.

Therefore, in order to analyze the relationships between the risk scores and the immune status, ssGSEA was applied for the purpose of quantifying the infiltrating scores of the different immune cell subsets (**Figure 6C**) and immune-related functions (**Figure 6D**) in the high- and low-risk groups. It was found that the regulatory T cell (Treg) and macrophages had increased infiltration in the high-risk group.

### Experimental Verification Processes

**Figure 7:**
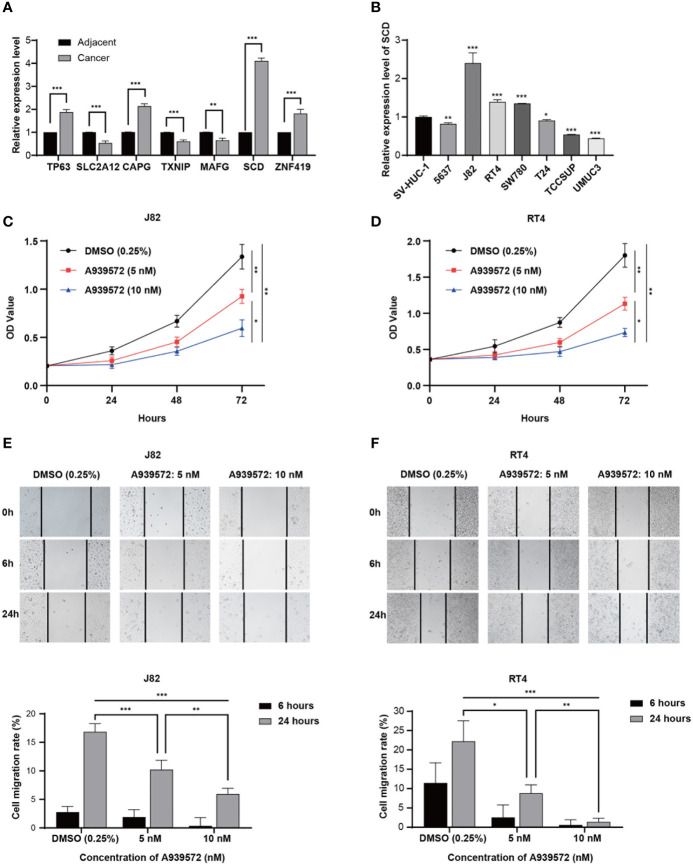
Experimental verification processes. **(A)** Marplot exhibiting the relative expressions of the seven genes evaluated by RT-qPCR in 16 bladder cancer samples and paired normal samples. **(B)** Relative expression levels of SCD in eight cell lines. **(C, D)** CCK-8 assay results show the relative proliferation of the J82 and RT4 cells after the addition of A939572. **(E, F)** Scratch assay results showed migration rates of the J82 and RT4 cells. In the figure, the data were shown as means ± S.D. *P < 0.05, **P < 0.01, ***P < 0.001.

In accordance with the above-mentioned results screened in the constructed model, RT-qPCR was performed in order to validate the expressions of the seven genes in the examined 16 bladder cancer samples and paired normal samples. As shown in **Figure 7A**, the TP63, CAPG, SCD, and ZNF419 were found to be significantly upregulated in the bladder cancer tissue samples. Meanwhile, the expressions of SLC2A12, TXNIP, and MAFG were observed to be markedly downregulated when compared with the adjacent normal samples, which was consistent with previous analysis results. In addition, SCD was chosen to further verify the accuracy of the model. The relative expression levels of SCD were validated in seven bladder cancer cell lines and one urothelial cell line by first conducting RT-qPCR, as illustrated in **Figure 7B**. The expression levels of SCD were found to be relatively high in the J82 and RT4 cells. Therefore, the J82 and RT4 cells were used to perform a proliferation assay. As detailed in **Figures 7C, D**, after inhibiting SCD activity by adding 5 nM or 10 nM of A939572, the cell growth was significantly decreased when compared to that of the DMSO (0.25%) control (23).

Next, a scratch assay was carried out in order to evaluate the effects of the A939572 on cellular migration. It was determined that the migration rates of the J82 and RT4 cells were markedly decreased in the cells treated with A939572 when compared with those treated with the DMSO (0.25%) control, as shown in **Figures 7E, F**.

## Discussion

Bladder cancer is a highly malignant form of cancer characterized with high morbidity and mortality, particularly in the cases of muscle-invasive bladder cancer (MIBC). MIBC generally has poor prognoses and high recurrence rates after the first resection. Several studies have suggested that a complex molecular biological link exists between bladder cancer cell proliferation and iron ions (24, 25). However, at the present time, the exact relationship between ferroptosis and bladder cancer has not been reported in detail. This study performed a screening process for genes associated with ferroptosis, which could potentially affect the prognoses of bladder tumor patients and constructed a prognostic model for the first time.

The seven DEGs which were identified to establish the FRPM were TP63, SLC2A12, CAPG, TXNIP, MAFG, SCD, and ZNF419. Among those, SLC2A12, TXNIP, and MAFG were found to have low expressions in the tumor tissue samples, while the others were highly expressed.

SCD is a critical enzyme in lipid metabolism, and its expression is correlated with the malignant transformation, proliferation, and survival of tumors (26–28). Huang et al. confirmed that SCD is highly expressed in lung adenocarcinoma and can promote tumorigenesis, cell migration, and invasion *in vitro* and *in vivo* (29). Du et al. reported that fibroblast growth factor receptor 3 (FGFR3) stimulated Stearoyl Coa Desaturase activity in order to promote bladder tumor growth. Furthermore, tumor progression was found to be substantially inhibited in a bladder cancer xenograft model after the knockdown of scd (30). A939572 is a novel and potent inhibitor of stearoyl-CoA desaturase (SCD) with IC50s of < 4 nM and 37 nM for mouse SCD and human SCD, respectively (23). In the present study, it was found that SCD was highly expressed in the examined tumor tissue samples. The inhibition of SCD by the addition of A939572 suppressed tumor proliferation and migration, which was consistent with the conclusions reached in previous studies.

The TP63 gene is one of the members of the TP53 family of tumor suppressor genes. The different protein isoforms play tumor suppressor and tumor-promoting roles in cell cycles and apoptosis (31–33). Guo et al. reported that TP63 mediated invasion in basal subtype bladder cancer cases using 3D invasion assays and orthotopic xenograft models through directly regulating the Ataxia-telangiectasia group D complementing gene (ATDC) expressions. ATDC is known to be highly expressed in human bladder cancer cases and promotes tumor formation and invasion (34).

The CAPG protein is a member of the gelsolin superfamily and participates in the remodeling of muscle fibers, as well as controlling such cellular behaviors as apoptosis and endocytosis. It is known to have an important role in rapid cell proliferation, local invasion processes, and distal metastasis. In addition, it has been demonstrated to be overexpressed in many types of tumor cells, including lung, pancreatic, breast, ovarian, melanoma, and glioma. Zhang et al. reported that CAPG promoted tumorigenesis and epithelial-mesenchymal transition *via* the Hippo signaling pathway in human bladder cancer cases (35).

Thioredoxin-interacting protein (TXNIP) is a thioredoxin-binding protein which can induce apoptosis and inhibit cell proliferation. It is considered to be a tumor suppressor in various cancer types. Koji Nishizawa et al.’s study suggested that TXNIP negatively regulated bladder carcinogenesis by suppressing ERK activation induced by the SDF-1-CXCR4 pathway (36).

In this research investigation, the univariate Cox regression analyses between the risk groups and the clinicopathological features showed that patients’ ages and the tumor stages strongly correlated with the survival status, while it was observed that the tumor grades did not display strong correlations with the survival status. However, according to previous studies and clinical practices, the grades of the bladder tumors were generally considered to have significant impacting effects on the prognoses. The main reason for the differences in this study’s results and the previous findings may have been that the sample size of the low-grade patients was only 21 patients and their survival states were that they were all alive. The uneven distribution of the samples in this study may have also potentially led to the Cox regression analysis not being fully completed by the “survival” R package.

Therefore, that part of the analysis was completed using SPSS software instead. The upper limit of the hazard ratio was even as high as 1899.358. However, that result could have been compensated for if more samples had been included.

The GO and KEGG analysis results suggested that the DEGs were associated with lipid metabolism and glutathione metabolism. At the present time, the mainstream view is that lipid chain oxidation is the direct cause of ferroptosis (37). Under the catalysis of iron ions, hydroxyl radicals undergo chain oxidation with unsaturated fatty acids, resulting in the destruction of membrane structures, production of aldehydes, and increases in membrane permeability, which finally leads to cell death (38). During this process, lipid oxidation and synthesis metabolism play essential roles (8). Glutathione peroxidase 4 (GPX4) reduces the peroxides in complex lipids (such as fatty acids, phospholipids, and cholesterol) in order to stabilize the hydroxyl lipids by consuming glutathione, thereby inhibiting the chain oxidation of cell membranes. Therefore, the GPX4 functions to scavenge lipid peroxides and inhibit ferroptosis, and glutathione is its obligatory cofactor (39).

The immune cell subsets and immune-related functions analyses showed Treg and macrophages had increased infiltration in the high-risk group. It is known that the Treg attenuates autoimmune and anticancer responses and plays an essential role in the immune escape of human malignant tumors (40, 41). Therefore, high frequencies of intratumoral Treg tend to suggest poor prognoses in various types of tumors, including ovarian cancer, pancreatic cancer, hepatocellular carcinoma (40, 42). Thomas Horn et al. found that tumor-associated antigen directed T cell responses were enhanced in a cohort of patients with metastatic bladder cancer by Treg depletion (43).

Tumor-associated macrophages (TAM) are associated with the infiltrative growth of tumors and are the most infiltrating immune cells surrounding the tumor tissue (44). In the study conducted by Qiu et al., it was revealed that tumor‐associated macrophages promoted bladder tumor growth through PI3K/AKT signals (45).

In summary, this study defined a novel prognostic model of seven ferroptosis-related genes which was proven to be independently associated with the OS of BLCA. A nomogram was developed to provide insight into the accurate predictions of BLCA prognoses. However, the specific mechanisms of the relationships between the ferroptosis-related genes and the prognoses of bladder tumors require further investigation.

## Data Availability Statement

The original contributions presented in the study are included in the article/**Supplementary Material**, further inquiries can be directed to the corresponding authors.

## Ethics Statement

The studies involving human participants were reviewed and approved by The Ethics Committee of Beijing Chao-Yang Hospital. The patients/participants provided their written informed consents to participate in this study.

## Author Contributions

XH and XW conducted this research study and revised the manuscript. MZ, XZ designed the research processes; exported the figures; wrote the first draft of the manuscript. MY performed the verification experiments. WZ analyzed the experimental data. DZ and SZ collected the clinical samples used in this research investigation. All authors contributed to the article and approved the submitted version.

## Funding

The present study was supported by the National Natural Science Foundation (grant no. 81970645) and the Beijing Natural Science Foundation (grant no. KZ202010025036).

## Conflict of Interest

The authors declare that the research was conducted in the absence of any commercial or financial relationships that could be construed as a potential conflict of interest.

## Publisher’s Note

All claims expressed in this article are solely those of the authors and do not necessarily represent those of their affiliated organizations, or those of the publisher, the editors and the reviewers. Any product that may be evaluated in this article, or claim that may be made by its manufacturer, is not guaranteed or endorsed by the publisher.
